# Symptoms Arising From the Diaphragm Muscle: Function and Dysfunction

**DOI:** 10.7759/cureus.53143

**Published:** 2024-01-29

**Authors:** Bruno Bordoni, Rohini Kotha, Allan R Escher

**Affiliations:** 1 Physical Medicine and Rehabilitation, Foundation Don Carlo Gnocchi, Milan, ITA; 2 Anesthesiology, H. Lee Moffitt Cancer Center and Research Institute, Tampa, USA; 3 Anesthesiology/Pain Medicine, H. Lee Moffitt Cancer Center and Research Institute, Tampa, USA

**Keywords:** cardio rehabilitation, physiotherapy, manual therapy, fascia, osteopathic, osteopathy, breathing, diaphragm

## Abstract

There can be many reasons that damage the function of the diaphragm, either transiently or permanently, involving one hemilate or both muscle portions. The diaphragm is associated only with breathing, but many other functions are related to it. The patient is not always aware of the presence of diaphragmatic dysfunction, and it is not always immediate to identify non-respiratory diaphragmatic symptoms. Pseudoanginal pain, night sweats, difficulty memorizing, or muscular and visceral problems of the pelvic floor are just some of the disorders linked to reduced diaphragmatic contractility. A decline in respiratory contractile force can be the basis for further pathological conditions that can increase the rate of mortality and morbidity. The article reviews the possible symptoms that may be presented by the patient, which are not necessarily related to lung function.

## Introduction and background

The diaphragm is the most important respiratory muscle and is involved in multiple bodily functions, such as coughing, sneezing, swallowing, vocalization, defecation, and urination [1]. It is fundamental for the function and circulation of the lymphatic system, the glymph, the cerebrospinal fluid, and the blood; it also plays a fundamental role in the perception of proprioception, pain, and emotional status [2-7]. The diaphragm is the postural muscle par excellence for maintaining balance, the stability of the lumbar area, and the expression of efficient neural coordination [8-10]. Breathing positively influences the cognitive aspect, stimulating synaptogenesis [10,11]. The diaphragm is innervated by the phrenic nerve and the vagus nerve (hiatal esophageal area); the origin and insertion of its contractile mass involve the xiphoid process and the last six ribs (posteriorly and superiorly). The right and left medial pillars (from which the intermediate pillars are formed) attach in the anterior area of the thoracolumbar vertebrae (T10-L4), while the lateral pillars make contact with L2 and the last rib [2,3]. The diaphragm separates the mediastinum from the abdomen, but it is a muscle full of passages for vessels (aortic hiatus, chyle cistern, arteries, vena cava, azygos, and hemiazygos veins) and for the passage of nervous pathways (sympathetic system, spinal system, parasympathetic system) [2,3].

There are many clinical areas where the diaphragm undergoes a non-physiological adaptation, which negatively impacts the patient's symptom picture. In stroke patients, there is a relationship between neuromotor weakness (gait, strength, balance) and the contractile expression of the diaphragm; the greater the weakness of inspiration, the greater the motor dysfunctions [12]. In patients with systemic lupus erythematosus, the diaphragm shows a general weakness (less excursion, less strength, less thickness), with a concomitant reduction in functional performance. Training aimed at the respiratory muscles allows you to improve the contractile expression of the diaphragm and the strength and coordination of the limbs [13]. Patients suffering from unilateral cervical radiculopathy (UCR) have a breathing pattern that is eupneic and under effort, non-functional, and with decreases in pulmonary parameters deduced from spirometry [14]. UCRs demonstrate an altered movement of the hemilate of the diaphragm corresponding to the side of the radiculopathy by fluoroscopic examination, with a reduced contraction capacity [14]. In half of patients undergoing mechanical ventilation (post-surgery, critical illnesses), they develop ventilator-induced diaphragm dysfunction (VIDD) within 24 hours of intubation; diaphragm atrophy is found, which increases the death rate and length of stay in the intensive care unit (ICU) [15].

People with obesity may suffer from obesity hypoventilation syndrome (OHS), a diaphragmatic weakness due to structural alterations such as excess accumulation of lipids with lipotoxic effects on contractile cells and reduction of lung volumes due to non-physiological positioning of the diaphragm caused by abdominal fat mass [16,17]. Chronic kidney disease (CKD) patients, with or without hemodialysis, demonstrate a reduction in diaphragm strength and thickness, whose alterations cause dyspnea, hiccups, and generalized fatigue [18]. One of the most probable causes of diaphragmatic atrophy is a systemic alteration of the renin-angiotensin system [18].

The diaphragm can suffer lesions due to paralysis of the phrenic nerve due to surgery, interscalene blocks, direct trauma to the chest, the presence of tumor masses, or systemic pathologies that damage the integrity of the nerve [19-23]. Despite these traumatic circumstances, the patient or clinician is not always aware of the existence of a lesion to the phrenic nerve, with paralysis of one side of the diaphragm (rarely bilaterally), whose symptoms are not immediately related to dysfunctional breathing [24]. The article examines the symptoms associated with a phrenic lesion and/or diaphragmatic weakness, which may be underestimated but can lead to additional pathological adaptations that adversely affect patient mortality and morbidity.

## Review

Functional diaphragm

**Figure 1:**
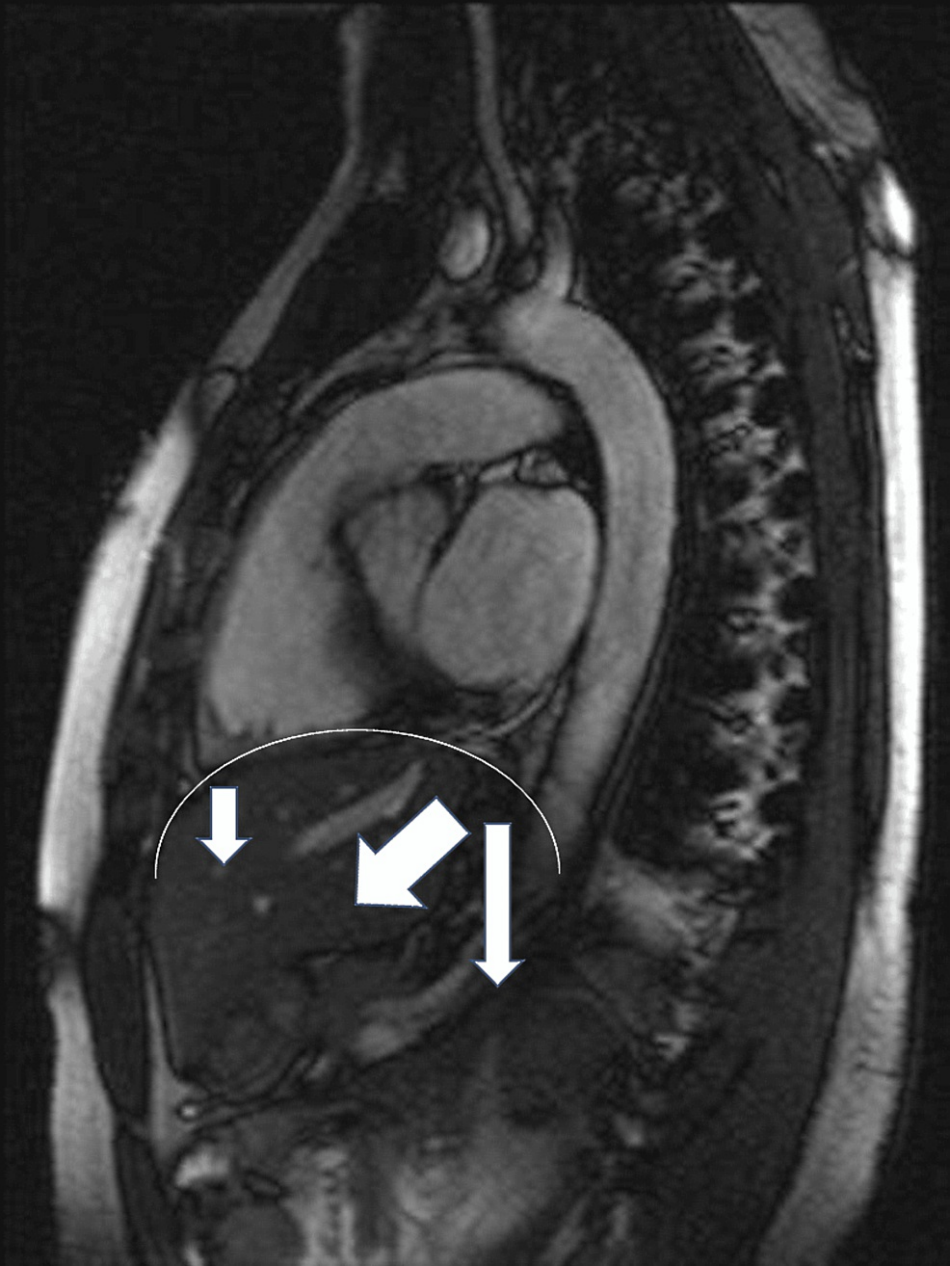
The image shows the position of the diaphragm in a healthy subject (curved white line). The examination is done with magnetic resonance imaging in the sagittal section. The white arrows indicate the direction of the diaphragm during any inhalation (oblique, anterior, and caudal). The image is the property of Bordoni Bruno (there is the patient's consent for the use of this figure).

Eupnea consists of a physiological breathing rhythm, with 12-20 breaths per minute in healthy adults [25]. During an eupneic breath, approximately 70% is carried out by the intervention of the diaphragm, with a speed of contraction and movement of 1.52 cm per second and an inhalation time of approximately 1.5 seconds and 3 seconds after adding the exhale [25,26]. An inhalation at rest can move with a range equal to or greater than 2 cm and a thickness equal to or greater than approximately 0.22 cm at the end of the inhalation; at the end of exhalation, the diaphragm has a thickness equal to or greater than 0.20 cm (Figure 1) [26].

Compared to the left dome, the right diaphragmatic dome is always higher by about 1.9 cm; however, at the end of a deep inhalation, the difference is reduced to approximately 1.5 cm. The right phrenic nerve has a faster speed of electrical impulses (per meter per second) than the left, as the right side is shorter (the left nerve rests on the left ventricle and is longer). This means that, despite the non-homogeneous height of the diaphragmatic domes, the different electrical speed compensates for the difference in position. The right diaphragmatic dome is faster in movement than the left area; the left diaphragmatic dome moves more slowly but with a smaller initial amplitude [10].

Maximal inspiratory pressure and sniff nasal inspiratory pressures

A physiological value of maximal inspiratory pressure (MIP), i.e., the strength of the inspiratory muscles, is 80 cmH_2_O [27]. With these parameters, the work of the diaphragm is not specifically identified but an intervention of the general inspiratory muscles. Another value to measure the functionality of the inspiratory muscles is the sniff nasal inspiratory pressure (SNIP). SNIP measures the force expressed at the esophageal level (sniff Poes), at the gastric level (sniff Pga), and the transdiaphragmatic pressure (sniff Pdi); although there is no real gold standard as reference parameters, a functional diaphragm is generally considered with values of 52-150 cmH_2_O sniff Poes, 68-62 cmH_2_O sniff Pga, and 82-204 cmH_2_O sniff Pdi [24,28].

The movement of the diaphragm is complex, involving multiple force vectors and multiple directions; the greatest excursion is due to the postero-lateral area (about 40% more than the antero-lateral area), with oblique antero-caudal movement during inspiration (Figure 1) [29,30]. Overall, the shape of the muscle does not change much during a respiratory act, as the major work is carried out by the peripheral diaphragmatic areas (costal area) [29].

Dysfunctional diaphragm

By taking a study of cardiac surgery patients and comparing the parameters of healthy subjects who did not undergo surgery, it can be deduced that a dysfunctional diaphragm has an inhalation excursion of less than 2 cm and parameters lower than those of a properly functioning diaphragm: an inhalation time of 0.93 seconds, an inhalation and exhalation time of 2.32 seconds, and a concentric contraction speed of 1.78 cm per second [26]. By averaging the thickness (thickening fraction, TF) in centimeters of the complete movement of the diaphragm (inhale/exhale), it is possible to have data on diaphragmatic function using ultrasound. A diaphragm with paresis could result in a TF below 20% compared to the healthy subject; an average of 20-36% compared to the TF of a healthy subject could be considered a dysfunctional diaphragm [31]. An MIP below 70 cmH_2_O (for females) reflects a dysfunctional diaphragm; for men, an MIP below 80 cmH_2_O is the result of a dysfunctional diaphragm [27]. There are numerous causes that induce a lesion of the phrenic nerve or weakness and which can involve different anatomical areas of the nerve path (medulla and outside the medulla), as well as the central nervous system, both for local or systemic pathologies and for direct or iatrogenic trauma; we recommend further study with the review by Ricoy et al. [32]. We do not have specific parameters in the literature for every pathological situation and every single clinical evaluation tool [25].

Diaphragmatic dysfunction due to phrenic nerve injury

Thoracic surgery can cause iatrogenic damage to diaphragmatic function due to damage to the phrenic nerve. The rate of post-cardiac surgery phrenic injury can reach 60% of patients [26]. Phrenic lesions are found after lung transplantation and after lung resection surgery, with a percentage that can reach 29.6% [33]. Although the patient may be in a cardiopulmonary setting, the clinician does not always guide therapeutic choices based on the evaluation of the diaphragm but rather on improving pulmonary respiratory function [34]. This often results in a limited result compared to expectations. Furthermore, diagnostic tools are not always effective in highlighting a phrenic weakness or lesion, as the examination does not always specifically take the diaphragm into consideration [34]. The most appropriate instrument or specialized clinician is not always available for a specific examination (electrical or magnetic phrenic stimulation, fluoroscopy, magnetic resonance imaging, computerized axial tomography, ultrasound) [34].

Morbidity and adaptation of the dysfunctional diaphragm

When the diaphragm does not function properly and chronically, the risks of developing pneumonia and myocardial infarctions increase [34,35]. Understanding whether there is a diaphragm dysfunction is clinically relevant.

Patients with undiagnosed unilateral diaphragm paralysis (UDP) or diaphragm muscle weakness often have no respiratory symptoms at rest [34]. This situation can be explained because some people use (unintentionally) the healthy side of the diaphragm in a major way to compensate for the dysfunctional side (increase in efficiency of activated motor units; increase in the number of recruited motor units); but this rule is not valid for all patients [24,36]. It can increase the activation of accessory respiratory muscles, such as the sternocleidomastoid, the scalene muscles, the external and parasternal intercostals, and the triangularis sterni muscle; these muscles can account for approximately 15% of transdiaphragmatic pressure in healthy subjects [36].

When patients are diagnosed as UDP in the absence of concomitant pathologies and subjected to tests (spirometry), they may present a restrictive pattern, but with healthy lungs, forced expiratory volume (FEV1) and forced vital capacity (FVC) are reduced compared to healthy subjects, regardless of the diaphragmatic side involved [24]. FVC has a greater decrease if the measurements are taken in the supine position; approximately 35% decline compared to normal values with subjects standing, and a further decrease of approximately 12% in the supine position [24]. Furthermore, they may have reduced total lung capacity (TLC), carbon monoxide diffusing capacity, and slight hypoxemia values [24]. There would appear to be a neuromechanical dissociation or dysphrenia: low tidal volume in the presence of a high respiratory rate [24].

Symptoms

Symptoms that can derive from the presence of a phrenic lesion can be orthopnea, often mistaken for the presence of cardiac and pulmonary pathologies, as well as dyspnea due to moderate efforts [37]. We may encounter sleep disorders, profuse night sweats, generalized tiredness during the daytime, sleep apnea syndrome, unidentifiable chest pain, and gastroesophageal reflux [37,38]. Let us remember that lung volumes cannot be used as a diagnosis to identify a phrenic lesion or a weakness of the diaphragm, as it is the strength of the diaphragm that is lacking, not a frank lung pathology [39]. Polysomnography can be used to get some indication of diaphragmatic respiratory problems; generally, UDP events such as central hypopnea can be recorded during the rapid-eye-movement (REM) sleep phase, with nocturnal desaturation events [32].

Hoover's Sign

It is possible to detect paradoxical movements (Hoover's sign) during a deep inhalation, but this does not always happen. Some diaphragmatic muscle adaptations may hide diaphragmatic weakness, a phenotypic change in the fibers of the healthy hemilate (more strength or more resistance), and/or the presence of fibrosis in the weaker hemilate, limiting the paradoxical respiratory movement [40]. Furthermore, even in some healthy subjects, paradoxical breathing may be present, despite the absence of pulmonary or phrenic pathologies [41].

Non-respiratory Symptoms

Let's remember that the neuroanatomical structure of the diaphragm allows the muscle itself to function as two muscles in one; each hemilate can work independently, and this complicates the differentiation between a healthy and dysfunctional diaphragm [42]. The primary motor cortex can send motor orders to the diaphragm towards the ipsilateral and contralateral sides; furthermore, the neurons of the phrenic nerve perform anastomoses for approximately 1-3% of the medulla before becoming roots, reinforcing the action of the electrical impulse [43].

By auscultating the lung bases, it is possible to find a reduction in vesicular murmur and dullness upon percussion, which signs could mimic the presence of pleural effusion [42]. Probably at night, considering that during sleep the diaphragm is involved as the main respiratory muscle for 81% compared to other muscles, these symptoms could be worse [34].

Diaphragmatic dysfunction or weakness could present as pain in the cervical spine and shoulder joints [32]. Possible signs of dysphagia include difficulty vocalizing, chronic cough, and trigeminal pain, the latter for the anastomotic connections with the phrenic nerve and the fifth, ninth, eleventh, and twelfth cranial nerves [34,44]. Pseudo-anginal pain, symptoms related to the thoracic outlet (paresthesia or pain in the upper limb), temporomandibular joint disorders, increased activity of the sympathetic system, alteration of the heart rhythm, blood pressure values, and venous return disorders. These clinical and symptomatic scenarios are linked to the anastomosis of the phrenic nerve not only with the cranial nerves but also with the sympathetic system (stellate ganglion) and the influence that breathing has on cardiovascular function [44-46]. A dysfunctional diaphragm could give symptoms of non-specific lower back pain, problems related to the sphere of the pelvic floor (muscles and visceral functions), in the absence of previous pathologies or traumas to the pelvic floor, and abdominal bloating; these problems can occur due to inadequate management and/or the creation of intra-abdominal pressures [1-3,6,34]. By decreasing its contractile efficacy, diaphragmatic dysfunction could cause an increase in lung infections and chronic atelectasis [34].

Other symptoms are linked to the strong relationship between the diaphragm and the vagus nerve, stimulating the spinosolitary pathway (proprioception stimulated by diaphragmatic movement) and the parasympathetic response that follows [47]. An alteration of the correct parasympathetic responses creates neuromotor coordination disorders, emotional disorders (anxiety, depression, stress), and a lowering of pain tolerance [4,5,7-9,34].

A dysfunctional diaphragm, following cardiovascular rehabilitation, could be further masked by the fact that the meters walked with the six-minute walking test are comparable with patients without respiratory dysfunction; what changes is the overall work, that is, a greater expressed fatigue and a smaller increase in metabolic equivalent of task (MET) at the end of rehabilitation [26].

A difficulty in mental attention - a decline in memory in the absence of brain pathologies - could be caused by a dysfunctional diaphragm [11,48].

**Figure 2:**
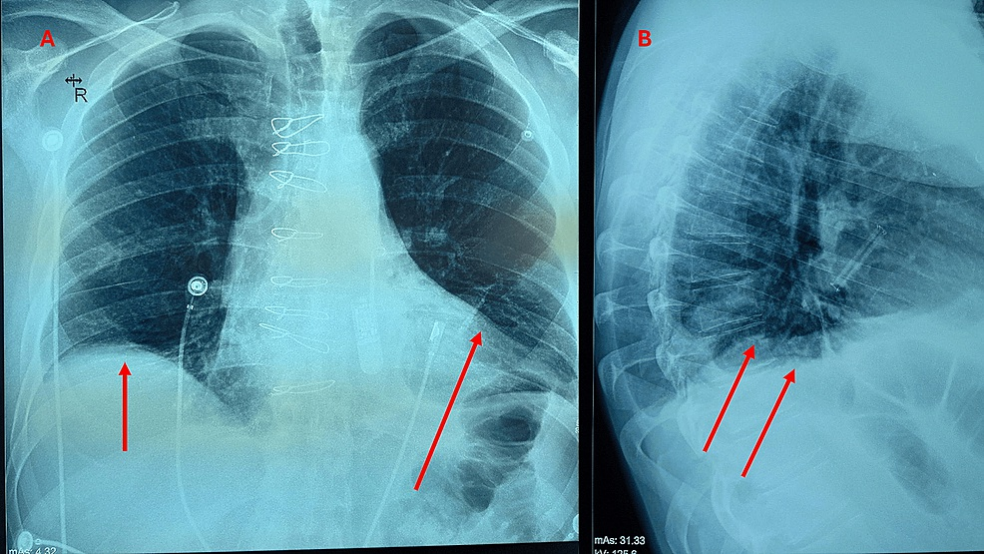
The X-ray shows in two different projections the elevation of the diaphragm bilaterally due to a bilateral lesion of the phrenic nerve in previous cardiac surgery with median sternotomy and pacemaker placement. The red arrows indicate the elevation of the diaphragmatic domes: (A) frontal plane and (B) sagittal plane. The patient presented with exertional dyspnea post-surgery, without the need for mechanical ventilation support. The image is the property of Bordoni Bruno (there is the patient's consent for the use of this figure).

Symptoms of bilateral injury are more intense in the respiratory area, with the possible need for mechanical ventilation support (cyanosis, superficial breathing, paradoxical movement); at rest, there may be no symptoms (Figure 2) [32,34,37].

Eventration

Another diaphragmatic dysfunction is the presence of eventration, a rare congenital or acquired condition, for which the adult patient may not present respiratory symptoms. Diaphragmatic eventration is the presence of extensive fibrosis compared to the muscle tissue; this anomaly represents approximately 0.05% of the population; the diaphragm is thinner, and the involved side(s) are elevated [32,48]. Symptoms, if present, are expressed as dyspnea, chest pain, recurrent pneumonia, gastroesophageal reflux, and palpitations. It can be confused with diaphragmatic hernia or paraoesophageal hernia [49].

Diaphragmatic Flutter

Another rare dysfunction affecting the diaphragm muscle is diaphragmatic flutter, repeated and involuntary contractions (exacerbated by stress), with pulsations noticeable in the epigastrium area, dyspnea, and abdominal and thoracic pain [32]. This dysfunctional form, also known as diaphragmatic myoclonus or tremor, or Leeuwenhoek disease, can be caused by central or peripheral lesions, but its specific etiology remains elusive [50]. There are no treatment guidelines but, rather, therapeutic subjectivation [32,50]. Furthermore, in the presence of pathologies or non-physiological conditions of the skeletal muscles, the diaphragm will always be negatively involved [42].

Diaphragmatic Hernias

Unlike congenital diaphragmatic hernias (Bochdalek and Larey-Morgagni hernia), which are often found in neonatal medicine and represent approximately 1-7% of the population, the symptoms of non-congenital diaphragmatic hernias (direct trauma that breaks parts of the diaphragm) they are more variable and dependent on the site of the muscle rupture and the size of the opening, not always easily identifiable and with incidental findings [2,3]. Some symptoms of diaphragmatic dysfunction that may occur are severe intestinal pain (volvulus, ischemia, obstruction) and immediate hospitalization, or simple digestive disorders. We have no data on the percentage of patients with diaphragmatic hernias related to trauma or iatrogenic damage due to previous abdominal or thoracic surgery.

Possible therapies

Considering the variety of causes that lead to diaphragmatic dysfunction, we have no information on the eventual recovery of contractility of the diaphragm and phrenic nerve.

The therapeutic approach is always subjective and based on different evaluations (chest X-ray, fluoroscopy, ultrasound, pulmonary function tests, stimulation of the phrenic nerve), based on the etiology and need. For a phrenic lesion, anti-inflammatory and bronchodilator drugs can be administered, although they are often ineffective [34]. A probable exception is theophylline (bronchodilator), as it seems to improve the contraction of the diaphragm muscle, but further data are needed [34].

If necessary, the clinician can use non-invasive positive pressure ventilation through different oral or nasal supports (continuous positive airway pressure, CPAP, bi-level positive airway pressure, BiPAP) [32,34]. Mechanical ventilation and the resulting invasive procedure are the last options for the instrumentation available to the clinician.

The plication of the diaphragm via a surgical approach is one of the most commonly used medical choices in the presence of a phrenic injury to improve the strength of the diaphragm and limit paradoxical movements [32,34]. Another surgical treatment in cases of phrenic injury is the reconstruction of the phrenic nevus, phrenic transplantation, the application of a pacemaker for electrical stimulation of the phrenic nerve, and the use of stem cells and growth factors (we have little data on the topic) [32,34].

The outcome of the chosen therapies will depend not only on the treatment imposed but also on the etiology and severity of the dysfunction itself.

## Conclusions

The diaphragm muscle is not only the main respiratory muscle but is also involved in multiple functions of the body system, such as the movement and stability of the trunk and limbs, vascular and lymphatic circulation, vocalization, and many other actions for the health of the body. If the diaphragm itself and/or the nerve conduction suffer damage, partial or complete, transient or permanent, not only its respiratory function but also the other functions assigned to it can be decreased. The symptoms in the presence of diaphragmatic or phrenic dysfunction are varied and variable and cannot always be immediately classified clinically; furthermore, the patient is not always aware of his own diaphragmatic contractile limitation. The clinician has numerous diagnostic tools and parameters at his disposal to understand if there is a functional diaphragmatic alteration; what is missing is the habit of adequately connecting the non-respiratory diaphragmatic functions to the patient's symptom picture. The article reviewed possible symptoms related to diaphragmatic dysfunction, phrenic injury, or contractile weakness.
